# Species distribution and antifungal susceptibility of *Candida* species causing candidaemia in a Thai university hospital during year 2006-2010

**DOI:** 10.1186/1753-6561-5-S6-P206

**Published:** 2011-06-29

**Authors:** K Ankkananukul, A Homkaew, P Chongtrakool, P Santanirand

**Affiliations:** 1 Department of pathology, Ramathibodi Hospital, Mahidol University, Bangkok, Thailand

## Introduction / objectives

Bloodstream infections due to *Candida* species play an important complications in severely-ill hospitalized patients which may lead to therapeutic failure. During a 5-year period from 2006-2010, 7 *Candida* species and 14 unidentified strains were identified from 887 candidaemia of patients admitted at Ramathibodi Hospital, Mahidol University, Bangkok, Thailand.

## Methods

*Candida* species were identified according to characteristics of chlamydoconidia production and carbohydrate assimilation/fermentation profile using 13/6 carbon substrates. Only a limited number of *Candida* were tested for antifungal susceptibility by agar gradient diffusion (Etest®)to amphotericin B and fluconazole.

## Results

From a total of 887 isolates, *Candida albicans* remains a major pathogen accounted for 50.3%, followed by *C. tropicalis*(27.4%), *C. parapsilosis* (12.5%), *C.guilliermondii* (6%), *C. glabrata* (1.8%), *C. krusei* (0.23%), *C. rugosa* (0.23%), and unidentified species(1.6%). From a total of 103 *C. albicans*, amphotericin B MIC ranged from 0.016-1ug/ml whereas fluconazole MIC ranged were between 0.023 to > 256ug/ml. Other species tested were *C. tropicalis* (73 strains) which yielded MIC range of 0.19-2ug/ml for amphotericin B and 0.5->256 ug/ml for fluconazole. 34 strains of *C. parapsilosis* gave amphotericin B ranged from 0.023-16ug/ml and 0.094->256 for fluconazole.

## Conclusion

*C. albicans* remains the most prevalence *Candida* species causing candidaemia followed by *C. tropicalis*, *C. parapsilosis* and *C. guilliermondii*. Those non-albicans *Candida* seemed to express decrease susceptibility to both major antifungal agents like amphotericin B and fluconazole whereas a small number of *C. albicans* gave high MIC to fluconazole.

## Disclosure of interest

None declared.

